# COVID-19: Are School Counseling Services Ready? Students' Psychological Symptoms, School Counselors' Views, and Solutions

**DOI:** 10.3389/fpsyg.2021.647740

**Published:** 2021-03-31

**Authors:** Mehmet Akif Karaman, Hasan Eşici, İsmail Hakkı Tomar, Ramin Aliyev

**Affiliations:** ^1^Department of Psychological Counseling and Guidance, Kilis 7 Aralık University, Kilis, Turkey; ^2^Department of Guidance and Psychological Counseling, Hasan Kalyoncu University, Gaziantep, Turkey

**Keywords:** COVID-19, psychological symptoms, high school students, school counselors, counseling services

## Abstract

The purpose of the current study was to investigate the effects of COVID-19 on high school students' psychological symptoms and to understand how ready counselors and school counseling services are based on the data we have. Therefore, this research is designed under two different studies: (A) Study 1: Effects of COVID-19 pandemic on students' psychological symptoms and (B) Study 2: Views and expectations of students and school counselors about school counseling services. The first study was a quantitative study and included 549 high school students (398 female, 151 male). A structural equation model (SEM) was created to examine the effects of COVID-19 pandemic on participants' psychological symptoms. The Impact of Event Scale-Revised (IES-R) scores showed that 107 (19.50%) individuals had a score of 50 and above pointing out that individuals in this group had severe impact of event/trauma symptomologies. The SEM analysis indicated that IES-R scores had a total effect of 0.79 on anxiety, 0.75 on depression, 0.74 on negative self-concept, 0.68 on somatization, and 0.66 on hostility scores. Furthermore, female students had significantly higher scores on anxiety, depression, negative self-concept, somatization, hostility, and impact of events variables than male students. Study 2 was a qualitative design and consisted of five school counselors and five students from different schools. The results indicated that students' difficulties during the coronavirus disease 2019 (COVID-19) outbreak were educational, cognitive, emotional, physiological, relational, technological, and related to routines. Academic, social, emotional, and behavioral issues came to the fore among the difficulties that can be experienced if students start face-to-face education. On the other hand, the school counselors listed that family relations, personal–social, emotional, and academic themes were the difficulties experienced by the students at the beginning of the COVID-19. In addition, when COVID-19 started, the services offered by school counselors were discussed under (1) services for the student, (2) services for the family, and (3) services for the teacher. Finally, according to the opinions of the school counselors, if students start face-to-face education, they may experience emotional, academic, and relational difficulties. In summary, it is vital that student personality services be prepared and implemented by school counseling services for schools based on the results.

## Introduction

In December 2019, the Chinese government was stirred by the findings of unexplained lung infections in four people working at a seafood and live animal market in Wuhan and in people who visited this market at the time (Evren and Us, 2020). On January 7, 2020, it was determined that a new type of coronavirus [severe acute respiratory syndrome coronavirus 2 (SARS-CoV-2)] was the basis of this disease (Xu et al., 2020) and since then, this virus has been defined as coronavirus disease 2019 (COVID-19) (WHO, 2020a). The WHO has declared a pandemic on March 11, 2020, and the first case was diagnosed on the same day in Turkey as well.

In the fight against coronavirus, due to the fact that the coronavirus is very contagious and the vaccine, which is the most important strategy in preventing the disease, was not yet available, the rapid and most effective implementation of other measures has come to the fore (Çöl and Güneş, 2020). In this context, many additional precautions have been taken around the world. The precautions first started with the ban on entering and exiting the city of Wuhan and continued with travel bans, curfews, country-based quarantine announcements, state of emergency announcements, prohibition of public events, and social isolation (Ministry of Health T. R., 2020; WHO, 2020b). One of the most important precautions taken in this regard was the interruption of education in schools in order to prevent students from becoming infected (Ali and Alharbi, 2020). Distance learning was introduced at all K-12 schools and at universities in Turkey as in many countries in the world (Reimers, 2020). In fact, in the 2020–2021 academic year, face-to-face education started with first graders, eighth graders, and high school 12th grades, but with the rapid increase in the number of cases (2nd Wave), schools were closed again, and full-time distance education continued. In this context, planned sports activities and all kinds of scientific events and artistic activities were postponed or canceled [Türkiye Bilimler Akademisi (TÜBA), 2020].

During the COVID-19 pandemic, students' access to education and training activities through online platforms has increased the impact of the crisis situation experienced. Social/physical distance and other limitations can cause negative psychological situations such as anxiety and fear, and these can affect students' well-being (Özer, 2020; Wang et al., 2020). Students have been shown among the groups with the most psychological difficulties during this period (Cao et al., 2020; Güngör et al., 2020) because they had to take part in distance education activities without participating in any orientation process and adapt to this process without support. This has created a general disappointment in students (Shores, 2020). Due to the difficulties experienced after COVID-19, children had difficulties in adapting to the situation and experienced learning difficulties, and this increased their anxiety (Çaykuş and Mutlu Çaykuş, 2020). Schools not only provide educational services but also many other services to students (such as food/nutrition, health care, educational programs, and mental health services).

The COVID-19 pandemic negatively affected the mental health and social, emotional, psychological, and educational well-being of young people (Golberstein et al., 2020). When other research results are examined, it has been stated that schools have various effects that can help alleviate this process in outbreaks (Sadique et al., 2008; Cauchemez et al., 2009; Demir Öztürk et al., 2020). It is normal for outbreaks to cause anxiety and fear in society (Güngör et al., 2020), but the anxiety and fear caused by the pandemics may make it difficult for individuals to combat the results of the outbreak. Identifying and responding to factors associated with anxiety can reduce individuals' anxiety levels and contribute to combating the pandemic (Banerjee, 2020; Brooks et al., 2020). In other words, the pandemic has brought not only the risk of illness and death but also negative psychological impacts (Duan and Zhu, 2020; Guo et al., 2020). For example, fear of getting sick, fear of being alone at home and delay in education, fear of families' inability to pay their education fees due to loss of income, and watching negative content on social media negatively affected the mental health of students (Kernan, 2019; Cao et al., 2020; Güngör et al., 2020; Tönbül, 2020). Increasing level of fear leads to a decrease in individuals' levels of well-being and hope. In addition, quarantine practices can increase the stress level and cause emotional problems (Fardin, 2020; Naeem et al., 2020; Qiu et al., 2020). The pandemic increases the risks of permanent mental distress (Liu et al., 2020). In the study conducted by YoungMinds (2020), 83% of young participants reported that the COVID-19 pandemic had worsened preexisting mental health conditions due to the closing of schools, loss of routine, and limited social connections.

The intensity and frequency of behavioral, cognitive, and emotional responses of students during the COVID-19 period can be reduced by suggestions given to families, teachers, and mental health professionals; behavioral and adaptational problems can be prevented or eliminated. Social support not only reduces psychological pressure during pandemics but also changes attitude toward social support and seeking help (Bai et al., 2005; Dorado et al., 2016). In a study conducted by Al-Rabiaah et al. (2020) on students during the Middle East respiratory syndrome coronavirus (MERS-CoV) epidemic, it was emphasized that students were experiencing stress and needed psychological assistance in such epidemics. Therefore, the need for mental health professionals (e.g., professional counselors) arises after situations, such as pandemics, natural disasters, and illnesses.

Udwin et al. (2000) found that students who received psychological support after the crisis were able to solve their problems in a healthy way and adapt to daily life more easily. During the previous SARS outbreak, some countries have opened online platforms to provide psychological counseling services to family members and other people affected by the pandemic and have identified methods for psychological crisis interventions that make it easier to cope with the impacts of the pandemic on public health (Duan and Zhu, 2020; Tönbül, 2020). Similarly, the Turkish Ministry of National Education has established a hotline for students and parents for psychosocial support. With the call of the Directorate General of Special Education and Guidance, professional counselors helped students and parents cope with the undesirable effects of COVID-19. In order to support students and their parents, 1,375 experts have been appointed to Guidance and Research Centers in all provinces of Turkey.

Changes in learning environments after the COVID-19 pandemic have created changes in the education process and practices. These changes affected all segments of the society as well as teachers. Teachers have tried to keep up with this new environment. At this point, school counselors also make extraordinary efforts to help students. However, since most of the job descriptions of counselors are formed around face-to-face contact, this new situation will be a process that needs to be explored for them as well. On the other hand, this will not be an obstacle for them, as school counselors make it their duty to help students and overcome obstacles (Kotarski, 2020).

In the SARS epidemic in 2003, it was reported that individuals' levels of knowledge, attitude, and panic played an important role in combating the disease (Person et al., 2004). In other words, experiences from previous outbreaks have revealed that individuals' knowledge, attitudes, and behaviors are important in controlling the spread of viruses (Johnson and Hariharan, 2017; Güngör et al., 2020). Even based on these researches, the support of counselors who have received professional training in the mentioned subjects in this process is important. In addition, counselors need to protect their mental health in order to help students. They should not neglect themselves. They should continue their activities that will be good for them (Kotarski, 2020).

The fact that the pandemic cannot be controlled in the COVID-19 outbreak and creates a wide sphere of influence and carries the risk of contamination to all people suggests that it will cause greater problems than other epidemics and pandemics. It is important to evaluate the educational/psychosocial effects as well as the health, economic, and political effects of the pandemic (Karataş, 2020).

When the literature is reviewed, it is seen that there are very few studies on adolescents during pandemic periods (Li et al., 2020). However, adolescence is a vital stage in development and emotional disorders, such as anxiety and depression can profoundly affect adolescents' health and school performance (Suldo et al., 2011). In this context, with COVID-19 pandemic in Turkey, a need has occurred for an evaluation of what role the counselors working at schools will play. Studies have highlighted many factors that contribute to the difficulties experienced by students. In addition, it is thought that there is much more to be learned about what can be done to reduce the negative psychological effects of the pandemic. More studies are needed to examine the impact of COVID-19 on students' mental health, and this should be done without delay. In this context, it is thought that this study will also shed light on this issue.

The purpose of the current study is to investigate the effects of COVID-19 on high school students' psychological symptoms and to understand how ready counselors and school counseling services are based on the data we have. Therefore, this research is designed under two different studies: (A) Study 1: Effects of COVID-19 pandemic on students' psychological symptoms and (B) Study 2: Views and expectations of students and school counselors about school counseling services.

## Study 1: Effects of COVID-19 Pandemic On Students' Psychological Symptoms

During the COVID-19 pandemic, students' access to education activities through online platforms has increased the impact of the crisis situation experienced. Social/physical distance and other limitations can cause negative psychological situations, such as anxiety and fear, and these can affect students' well-being. High school students are among the groups with the most psychological difficulties during this period (Campbell, 2020; Çaykuş and Mutlu Çaykuş, 2020). Because they have had to take part in distance education activities without participating in any orientation process and adapt to this process without support. Therefore, for the purpose of Study 1, the answers to the following research questions were examined:

1. What is the total effect of event (COVID-19 pandemic) on high school students' symptoms of depression, anxiety, negative self-concept, somatization, and hostility?
2. Are there significant differences between male and female high school students' scores of psychological symptoms and impact of event?

### Material and Methods

#### Power Analysis

To determine the number of cases needed for research question 1, at least five cases per parameter were calculated as a general rule of thumb (Brown, 2006). Considering that there were 75 parameters in the study, it was thought that a minimum of 375 participants would be sufficient for SEM analysis. An *a priori* power analysis using G^*^Power 3.0.10 (Faul et al., 2009) was used to calculate the minimum sample size needed to evaluate the research question 2. Using a high level of power of 0.95, a medium effect size as *f* ^2^ = 0.15, and a 0.05 alpha level (Cohen, 1992), the target sample size for this study was reported to be 146.

A total of 583 individuals participated in the study. Of them, 34 (5.83%) did not finish the entire questionnaire or failed to fill out two bogus items (check attention questions). The final sample included 549 individuals. There were more female (*n* = 398, 72.5%) than male participants (*n* = 151, 27.5%); the average age of participants was 16.1, SD = 1.01. In terms of grade levels, 146 students were in 9th grade, 176 were in 10th grade, and 227 were in 11th grade.

#### Procedure

After getting the Ethics Board approval from the relevant university, we created an online form via Google Forms. The study was conducted between May 15 and 25, 2020, when the quarantine restrictions were effective for individuals who were under 20 and above 65 years old. The online link was sent via emails to 10 high school principals who were randomly selected and represented seven regions of Turkey. There were 6,430 registered 9–12 grade students in these schools. Six school principals responded our emails and accepted to distribute the link to their students. We did not include 12th grade students in the study since they would graduate and not be in schools in the next academic term. Hence, the link was delivered to 2,893 students who were in 9–11 grades. Taking this number into account, we have reached a 20% response rate in the current study.

#### Measures

##### Brief Symptom Inventory

We used the Brief Symptom Inventory (BSI) Turkish form (Sahin and Durak, 1994) for the current study. Derogatis (1992) developed the original BSI based on the Symptom Check List-90. This 53-item instrument consists of five subscales evaluating anxiety, depression, negative self-concept, somatization, and hostility in the Turkish version. The BSI uses a five-point Likert-type response format with values ranging from 0 = *Not at all* to 4 = *Extremely*. There is also an R = Refused option if participants do not want to respond to the items. The BSI starts with the question of “DURING THE PAST SEVEN DAYS, how much were you distressed by:” and includes items such as “Thoughts of death or dying” and “Feeling lonely.”

Sahin and Durak (1994) reported moderate Cronbach's alpha coefficients for depression (α = 0.88), anxiety (α = 0.87), negative self-concept (α = 0.87), somatization (α = 0.75), and hostility (α = 0.76). For the current study, we calculated Cronbach's alpha coefficients for depression (α = 0.91), anxiety (α = 0.89), negative self-concept (α = 0.89), somatization (α = 0.82), and hostility (α = 0.82).

##### Impact of Event Scale-Revised

The Impact of Event Scale-Revised (IES-R) (Weiss and Marmar, 1997) was developed to assess impact of events and traumatic stress symptoms based on the Diagnostic and Statistical Manual of Mental Disorders IV-TR (American Psychiatric Association, 2000). In the current study, we used the IES-R Turkish form, which was validated by Çorapçioglu et al. (2006).

This 22-item instrument consists of three subscales assessing avoidance, intrusion, and hyperarousal. The IES-R uses a 5-point Likert-type response format with values ranging from 0 = *Not at all* to 4 = *Extremely*. The measure starts with the question of “DURING THE PAST SEVEN DAYS with respect to_______________, how much were you distressed or bothered by these difficulties?” and includes responses such as “I was aware that I still had a lot of feelings about it, but I didn't deal with them” and “I avoided letting myself get upset when I thought about it or was reminded of it.” An optimal cutoff score of 50 is used to diagnose posttraumatic stress disorder symptomology in community samples (Creamer et al., 2003). Wang et al. (2020) reported that a cutoff score of 37 or above could be used for “severe psychological impact” category. Çorapçioglu et al. (2006) reported a strong Cronbach's alpha coefficient of 0.94. For the current study, similar to the previous studies, we calculated a Cronbach's alpha of 0.92 as an indicator of strong consistency.

#### Statistical Analysis

**Figure 1:**
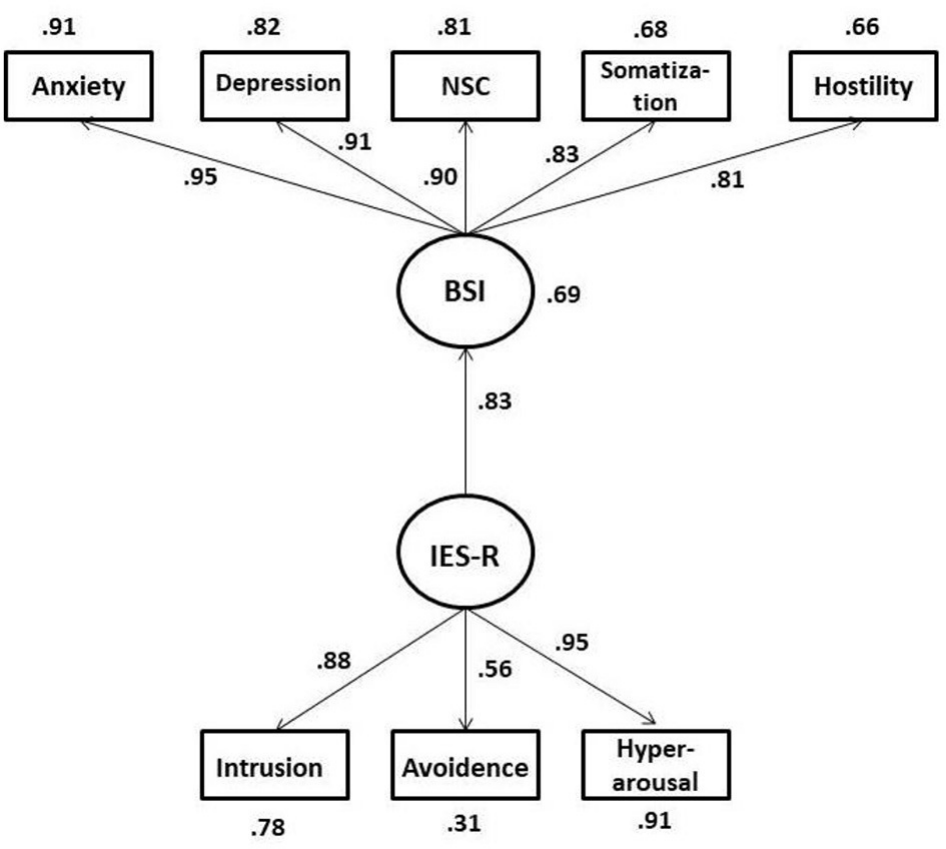
Structural model depicting the effects of Impact of Events Scale-Revised (IES-R) scores on Brief Symptom Inventory (BSI) Scores.

The SPSS 25.0 and AMOS 22.0 programs were used to run analyses. First, descriptive statistics, correlational analysis, and alpha coefficients were computed for each instrument used in the study (see Table 1). Next, an SEM was created to answer the research question one (see Figure 1). The assumptions of multivariate normality were tested. To assess the assumption of multivariate normality, the Mardia's statistic was computed. The results indicated that Mardia's coefficient was significant (8.66) and greater than the critical ratio (1.96; Mardia, 1970). However, this test is highly sensitive to larger sample size. Therefore, the kurtosis values for individual variables were inspected (Stevens, 2009), and all of the values were between −0.34 (anxiety) and −0.97 (depression), which means that the data were normally distributed (Westfall and Henning, 2013). We interpreted the chi square statistic (χ^2^) and *p-*values, as well as goodness of fit index (GFI), comparative fit index (CFI), Tucker–Lewis index (TLI), standardized root mean square residual (SRMR), and the root mean square error of approximation (RMSEA) metrics of model fit. We used the values for the χ^2^ (*p* > 0.05), GFI > 0.90, CFI > 0.90, TLI > 0.90, SRMR < 0.08, and RMSEA < 0.08 representing an acceptable model fit (Dimitrov, 2012).

**Table 1:** Means, standard deviations, reliability coefficients, and bivariate correlations for scores among variables.

**Variable**	**1**	**2**	**3**	**4**	**5**	**6**
1. Anxiety	–					
2. Depression	0.85^*^	–				
3. Negative self-concept	0.85^*^	0.85^*^	–			
4. Somatization	0.80^*^	0.73^*^	0.72^*^	–		
5. Hostility	0.76^*^	0.74^*^	0.75^*^	0.66^*^	–	
6. Impact of event	0.73^*^	0.67^*^	0.67^*^	0.64^*^	0.62^*^	–
*M*	17.76	20.53	15.90	9.02	11.28	31.11
*SD*	11.88	12.74	11.63	7.41	6.71	19.44
α	0.89	0.91	0.90	0.82	0.82	0.92

* *p < 0.01*.

To answer research question 2, a one-way multivariate ANOVA (MANOVA) was conducted. The independent variable was gender, and dependent variables were depression, anxiety, negative self-concept, somatization, hostility, and traumatic stress symptoms. We did not perform a *post-hoc* analysis since gender has two levels (male and female). Their mean scores on the scales were compared if the results indicated that there were significant differences.

### Results

The descriptive statistics and correlations between variables are presented in Table 1. The IES-R scores showed that 201 (36.6%) individuals had scores of 37 and above, indicating high impact of event. Of this, 107 (19.50%) had a score of 50 and above, pointing out that individuals in this group had severe impact of event/ trauma symptomologies. All the correlations among variables were significant. The impact of event (COVID-19 pandemic) had the highest relationship with anxiety (*r* = 0.73) and the lowest relationship with hostility (*r* = 0.62). The hypothesized model was tested, and the fit indices indicated that the model had a strong fit: χ^2^(19) = 71.98, *p* < 0.001, χ^2^/df = 3.78; CFI = 0.98, TLI = 0.98, GFI = 0.96, RMSEA = 0.07 (90% CI = 0.054–0.089), and SRMR = 0.022. All the regression weights were significant, *p* < 0.001. There was a strong direct effect of impact of event (COVID-19 pandemic) on psychological symptoms (0.83). This means that as the individuals' IES-R scores increased, their psychological symptom scores also increased. In addition, the results indicated that IES-R scores explained 69% of total variance in psychological symptoms of individuals. Specifically, IES-R scores had a total effect of 0.79 on anxiety, 0.75 on depression, 0.74 on negative self-concept, 0.68 on somatization, and 0.66 on hostility scores. In other words, individuals' traumatic-/pandemic-related events had high effects on their psychological symptoms when the data were collected.

A one-way MANOVA was conducted to answer research question 2. The analysis examined the effect of gender (male and female) on levels of anxiety, depression, negative self-concept, somatization, hostility, and impact of events. An alpha level of 0.05 was utilized. Assumption for homogeneity of covariance (Box's *M* = 6.98, *p* < 0.001) was not met. Therefore, Pillai's trace was reported instead of Wilks' lambda, since it was more robust than the other statistics to violations of model assumptions (Olson, 1974). A statistically significant effect was identified between gender and the six dependent variables, Pillai's *V* = 0.097, *F*_(6, 542)_ = 9.70, *p* < 0.001. Approximately 10% of the variance in the model was accounted for in the combined dependent variables across gender, yielding a small effect.

Effects among subjects were inspected, and it was found that there was a statistically significant difference between groups in terms of anxiety, depression, negative self-concept, somatization, hostility, and impact of events variables. There were statistically significant differences between male and female subjects through all independent variables. Based upon these results, estimated marginal means were reviewed (see Table 2) and found that female high school students had higher scores on all variables than male high school students.

**Table 2:** Descriptive statistics for male and female participants.

**Variable**	**Gender**	***M***	***SD***	***N***
Impact of event	Female	32.46	18.85	398
	Male	27.53	20.55	151
Anxiety	Female	18.92	12.09	398
	Male	14.68	10.76	151
Depression	Female	22.41	12.71	398
	Male	15.57	11.46	151
Negative self-concept	Female	16.68	11.94	398
	Male	13.86	10.56	151
Somatization	Female	9.54	7.48	398
	Male	7.63	7.09	151
Hostility	Female	11.63	6.78	398
	Male	10.37	6.44	151

### Discussion of Study 1

The main purpose of the Study 1 was to understand how COVID-19 pandemic affected Turkish high school students in the current study during COVID-19 quarantine restrictions. All school settings including K-12 and universities were closed, and individuals under 20 years old were banned to go outside their homes based on these quarantine measures. The findings of Study 1 indicated significant results of psychological symptoms of participants.

First, of the participants, ~20% (*n* = 107) reported a score of 50 or above on the IES-R scale. This scale measures the stress of individuals experiencing any trauma when the scale is applied (Çorapçioglu et al., 2006). Based on the result, a significant portion of participants showed traumatic symptoms during the quarantine restrictions. This finding is consistent with the previous studies just conducted in 2020 in other countries and cultures, stating that COVID-19 is an unusual, unexpected and traumatic event affecting individuals and societies in a wide range (Brooks et al., 2020; Demertzis and Eyerman, 2020; Fardin, 2020; Lei and Klopack, 2020; U-M Department of Psychiatry, 2020; Wang et al., 2020). Brooks et al. (2020) reviewed 24 studies that reported the psychological effects of pandemics on people. Symptoms of posttraumatic stress disorder, blurred mind, and anger were the risk factors reported most in the studies. One of the reasons for the current traumatic symptoms may be the feelings of insecurity and loss of control that participants experience during the quarantine process, as well as fears of losing family members and the people they know (e.g., friends, neighbors). Another reason could be the loss of routine and feeling out of control (Campbell, 2020). The COVID-19 has been an ongoing process, and little was known about it when the data were collected. As in many countries, regulations and sanctions have been introduced in Turkey to change daily life and habits. Wang et al. (2020) stated that being a student was associated with higher scores of IES-R. High school students could not attend their schools, had difficulty motivating their classes, could not go out, and when they left, they were fined. As a result, individuals went into a process during which they could not control themselves and moved away from their daily routine, which may have triggered trauma symptoms.

The results also showed that traumatic symptoms affected participants' psychological symptoms strongly. The IES-R scores had high total effects on anxiety, depression, negative self-concept, somatization, and hostility. Based on this finding and correlational analysis, as trauma symptoms related to COVID-19 pandemic increased, psychological symptoms increased as well. Feeling unsafe, fear of death, risk of getting the coronavirus, being away from the school and social relationships, prolonged home quarantine, uncertainty about the future, and financial difficulties experienced by families may have triggered individuals' psychological symptoms. For example, Wiederhold (2020) examined high school students' “likes” and “shares” on the social media platforms, such as Instagram, Snapchat, Reddit, and TikTok, and found that their social media shares were related to anxiety, depression, and feeling powerless. In a study conducted in China at the beginning of the pandemic (Liu et al., 2020), the researchers found that the pandemic increased the risks of permanent mental distress. In another study conducted by YoungMinds (2020), 83% of young participants reported that the COVID-19 pandemic worsened preexisting mental health conditions due to school closure, loss of routine, and limited social connections. Considering that 201 participants (36.6%) had a score of 37 or above (severe psychological impact) in the current study, COVID-19 pandemic process affected high school students depression, anxiety, negative self-concept, somatization, and hostility.

The last finding worth discussing is the differences between the male and female high school students' scores of psychological symptoms and impact of event. The results indicated that female students had higher scores on anxiety, depression, negative self-concept, somatization, hostility, and impact of event. In other words, female students experienced this process more severely than male students. The study of Wang et al. (2020) with participants with an average age of 21 in February, at the beginning of the corona, in the days when restrictions began in China, also found differences in psychological symptoms between men and women. This finding was consistent with previous studies. For example, Hou et al. (2020) found that female subjects had more severe stress and anxiety symptoms than male subjects. However, there was no difference on depression levels. A recent report released by Statistics Canada (Moyser, 2020) showed that female participants reported worse mental health than male participants since the COVID-19 restrictions began. Similar results were reported by the National Center for Health Statistics (Terlizzi and Villarroel, 2020) stating that female subjects' symptoms of anxiety disorder and depression were higher than that of male subjects. However, in another study conducted by Cao et al. (2020), gender did not have significant effect on anxiety. As can be seen from these findings, the effects of the COVID-19 pandemic on gender vary according to the participating groups. In the current study, girls have higher scores than boys, which may be due to the girls taking on other responsibilities (e.g., take responsibility for younger siblings, help with household work) as well as their lessons during the restrictions. Another reason could be the social support that female participants receive from their social relationships outside the home. As stated by Siddiqui et al. (2019), female participants receive higher social support from significant others and friends than male participants. In this context, female participants may have been more exposed to the psychological effects of pandemic because they were deprived of social support, which is one of the important sources of support.

In the next study, we explored the difficulties students experienced, their expectations when the schools started, and school counselors' readiness. Moreover, we tried to understand if the school counselors had a plan to help students cope with psychological symptoms.

## Study 2: School Counselors' and Students' Views On Difficulties Due to COVID-19

The aim of the second study was to determine the difficulties experienced by students due to COVID-19. For this purpose, online semistructured interviews were conducted with the students and the school counselors. In this section, information about the process and preliminary results obtained within the scope of the study were shared.

### Method

In this part, the difficulties experienced by the students due to COVID-19, the needs arising due to these difficulties, and the readiness levels of the schools to meet the students' needs were described with the phenomenological pattern, one of the qualitative research designs. The phenomenological approach mainly focus on the meaning, structure, and essence of the lived experiences of a phenomenon for a person or group of people (Patton, 2015). In this study, “the situation of schools in meeting the needs arising due to the difficulties experienced by students during the COVID-19 pandemic” was considered as a phenomenon and analyzed in depth. Answers to the following questions were sought: What kind of difficulties do students have in the pandemic process? What are their needs to deal with these challenges? After the pandemic, how ready are schools to respond to students' needs?

#### Participants

In accordance with the purpose of the study, participants were selected among the students and school counselors at high school level in Turkey using the maximum variation sampling method. In order to obtain more detailed information about the phenomenon examined, maximum diversity was achieved by taking into account studying or working in different cities and different types of high schools. In the interviews with the students and the school counselors, the differences were primarily determined, and then, a list of similar or common aspects among the differences was created. Five school counselors from different cities and five students registered at the schools of these counselors participated in the study. Three of the students were male and two were female. Two students (one female and one male) were in the 9th grade, two students (one female and one male) were in the 10th grade, and finally, one male student was in the 11th grade. Considering the demographic characteristics of the counselors, four were women and one was a man. The age of counselors varied between 39 and 44. Counselors have been in the profession for a long time, and they were experienced (varied between 17 and 21 years).

#### Measures

The data were collected using the semistructured interviews used in qualitative research designs. “Student and School Counselor Semi-Structured Interview Forms” were prepared to collect data. In the preparation of the interview question forms, first, researchers and field experts were interviewed, and a draft form was created. After the draft was examined by a researcher who is a faculty member in the field of counseling and who is an expert in qualitative research, a pilot study was carried out. In the pilot study, a school counselor and a high school student were interviewed, and the form was finalized by ensuring consistency for the interviews. The sample questions in this interview form are as follows: “What kind of difficulties did you have at the beginning of COVID-19?” (student question) and “When you return to the school environment for education; what kind of difficulties may your students experience?” (school counselor question).

#### Procedure

Before conducting the interviews, informed consent form was obtained from the students, their parents, and the school counselors. In addition, before the interviews, questions were sent to the participants for review. Appointments and interviews were made according to the appropriate days and times for the participants. All of the interviews were conducted online due to the risk of COVID-19 contamination. Interviews ranged from 25 to 57 min. The statements of the individuals interviewed during the interviews were repeated, and participant confirmation was obtained again.

#### Analysis

The data obtained from the interviews were analyzed in four stages in accordance with the purpose of the research and the phenomenological pattern determined. These stages consist of (1) coding of data, (2) finding themes, (3) organizing codes and themes, and (4) defining and interpreting the findings (Creswell et al., 2007). The data obtained from the interviews were coded under three topics: “difficulties” that students experienced at the beginning of COVID-19 and when they return to school, “assistance needs” that arise due to these difficulties and “readiness levels” of school counseling services, and relevant categories were created. Then, themes and subthemes were created. In this context, the audio recordings obtained from the interviews were first deciphered by a researcher who was competent and experienced in the interview technique in the research team and transformed into a written text in a Word document. Before proceeding with the content analysis of the written texts, the content analysis was started by ensuring the coder reliability between the two researchers determined for content analysis. Themes, subthemes, and codes were created as a result of the analysis. The obtained codes were examined by the research team, the themes and codes were given their final form, and the findings of the research were defined and interpreted.

##### Trustworthiness

In this study, which was carried out with the semistructured interview technique, in order to ensure trustworthiness, the participants were informed about the purpose of the study and the interview process, and consent was obtained from the participants to express themselves sincerely with voluntary participation. There are four criteria for trustworthiness (Mabuza et al., 2014): (1) credibility, (2) transferability, (3) dependability, and (4) conformability. To achieve credibility, the interview recordings transmitted to the text were sent to the participants. By taking the feedback on the text, errors were extracted; thus, the accuracy and precision of the information were checked. Second, to achieve transferability, we used maximum variation sampling method.

In order to ensure reliability in the interviews, it was carried out by a single researcher who was competent and experienced in qualitative research and interview technique. The meanings of the questions for the interviewer and the participants were tested by conducting a pilot study with an interviewer student and a school counselor with the interview protocol prepared before the interviews. In addition, in order to ensure consistency in transcribing the audio recordings obtained as a result of the interview, a part of the recorded interview was analyzed in two different times, and the consistency in both decoding processes was tested. To achieve dependability and confirmability, constant comparison was made to ensure reliability in the coding process of the categories. With this method, interview data were encoded in categories by two different researchers were compared with each other (Mabuza et al., 2014).

### Results

#### Findings on Students

We reported the difficulties experienced by students during the first semester break of face-to-face education at the beginning of COVID-19 in Turkey and the needs that arise due to these difficulties below. In addition, the difficulties they may face if they can start their face-to-face education again, the findings regarding the new needs that may arise based on these difficulties, and the readiness of schools to meet these needs of students are given below, respectively.

##### Difficulties Faced by Students During the Break in Face-to-Face Education at the Beginning of COVID-19

According to the findings of the interviews with high school students, the difficulties experienced by the students are divided into themes of educational, cognitive, emotional, physiological, relational, and technological difficulties and those related to routines.

*Educational Difficulties.* The most emphasized theme among the difficulties experienced by the students was related to their educational characteristics. The codes in this theme included preparation and participation in online lessons, time management in distance education, efficient studying, following courses, loss of academic achievement, online connection quality, staying away from educational materials, and weakening of school connection. It was observed that some of the students lost their connection and communication with the school during this process. A participant summarizes the situation, “*The school did not communicate with us much during this period. I couldn't feel the school with me*” (male, ninth grade student).

*Cognitive Difficulties.* While expressing the cognitive difficulties, findings were obtained regarding the participants' understanding of COVID-19 and realizing its seriousness. One participant expressed the process of making sense of COVID-19 and realizing its seriousness as “*At first, we thought it would last for a short while. Later, when the holiday got longer, we started to understand how serious the situation was*” (female, 10th grade student).

*Emotional Difficulties.* Among these difficulties, contagion anxiety, fear of death, fear of losing loved ones, fear of uncertainty, helplessness and sadness, quarantine pressure, and restriction of freedom were feelings experienced by some students. One participant expressed his opinion on the quarantine pressure as follows: “*I sometimes got angry because I stayed at home all the time*” (male, 10th grade student).

*Physiological Difficulties.* Among the physiological difficulties expressed by the students, there were sleep problems, appetite or overeating, sports activities, and face-to-face games. When the statements of the students were examined, the statement of a participant about difficulty in sleeping, which is chosen as an example of the situations they experience, was as follows: “*I couldn't open my eyes in the lessons. I've been sleeping and waking up even during the 10-minute break between classes*” (male, ninth grade student).

*Relational Difficulties.* Participants expressed their difficulties in relation to loss of relationship (friend, relative), loss of close contact, conflict with parents, sibling conflict, and visiting relatives. One of the participants expressed the relationship loss as follows: “*The most upsetting thing was the inability to communicate with my relatives. I really miss them*” (female, 10th grade student).

*Technological Difficulties.* Among the technological difficulties experienced by high school students were the problem of access and connection loss of online activities. A participant who expressed the problem of access: “*I work from sources myself, I try to find and watch new videos. This is getting a little more difficult. We could not get efficient lessons because the teachers had internet connection problems*” (female, ninth grade student).

*Routines.* It seems that some of the difficulties experienced by some students at the beginning of COVID-19 include changes in their routines. In situations that create anxiety and stress, routines may change during periods of uncertainty. Sleep time and meal time are among the routine changes experienced by the participants. One participant's views on the changes in sleep time were as follows: “*In the past, our lives were regular, we had a planned life. We were getting up at 8 in the morning and going to our school. Now our routine is broken*” (male, 10th grade student).

##### Difficulties That Can Be Faced if Students Start Face-to-Face Education

Academic, social, emotional, and behavioral themes come to the fore among the difficulties that can be experienced if students start face-to-face education.

*Academic Difficulties.* Participants expressed the issues of learning loss, loss of motivation, adaptation to the school environment, transition to face-to-face education, public places, education with masks, healthy transportation to school, and planning learning activities among the academic difficulties they may experience if they start face-to-face education. One participant expressed the learning loss with the following statements: “*We may not have learned as much from online education as we did from face-to-face training. Some of us may be behind the class*” (female, 10th grade student).

*Social Emotional Difficulties.* If they start face-to-face education, the students expressed the social and emotional difficulties they may experience as the fear of contagion, fear of loss of friends, insecurity about hygiene, worry that peers will be harmed, establishing intimacy, and maintaining relationships at physical distance. Expressing his fear of contamination, a student said, “*I am afraid that some of our friends will not keep their distance. If they get close to us then it will be dangerous*” (male, ninth grade student).

*Behavioral Difficulties.* Students stated that if they start face-to-face education, they will experience behavioral difficulties in physical contact, not touching things at school, and obeying the rules. One participant, on the other hand, stated the difficulty of obeying the rules, “*I think that students will not be able to take adequate precautions against the risk of virus in classrooms, and not everyone will follow the rules*” (female, ninth grade student).

##### Psychological Needs That May Occur for Students After COVID-19

If the students can start face-to-face education, they have certain expectations from the counseling service so that they can cope with the difficulties they may experience during the distance education process and in the process of readaptation to school. These expectations were divided into motivation, adaptation to school, peer support, determination of needs, social assistance, coping with the pandemic, individual support, planned work, and problem solving. A participant who wanted the counseling service to do an orientation program said the following: “*I want them to think about how they will enable us to adapt to the new situation and make preparations*” (male, 11th grade student).

#### Findings Regarding the Views of School Counselors

This section includes findings from interviews with school counselors who work at high schools in Turkey. The findings related to the difficulties experienced by students at the time of interruption of education at the beginning of COVID-19, services provided to the students in this process, the difficulties students can experience if they start face-to-face education again, and the planned services to be offered in Turkey will be cited.

##### Difficulties Experienced by Students at the Beginning of COVID-19

According to the analysis results obtained from the interviews with the school counselors, the difficulties experienced by the students at the beginning of COVID-19 were grouped under family relations, personal–social, emotional, and academic themes.

*Family Relations.* According to the views of school counselors, family pressure, family conflict, high expectations of family, conflict with parents, and protection of borders in family relationships were among the difficulties students experienced in family relationships. Stating that the expectations of families at the onset of COVID-19 were perceived as a problem by students, one participant expressed her opinion as follows: “*Families want their children to study as they did when the school was open*” (female, 17-year experience).

*Emotional Difficulties.* School counselors expressed uncertainty, fear of transmission, anxiety and restlessness, loneliness, boredom–depressed, death of the loved ones and mourning, and fear of losing loved ones among the emotional difficulties experienced by students at the onset of COVID-19. A participant stated for the students experiencing sense of grief, “*The father of one of our students, grandfather and sister living in the same apartment were tested positive for COVID-19. I had one-on-one meetings and studied with him. In fact, that student lost his grandfather*” (female, 19-year experience).

*Personal Social Difficulties.* Among the results obtained from interviews with school counselors regarding personal social difficulties were relationship building, autonomy, fun, responsibilities, communication conflict, and daily life skills. One participant told about the students who could not meet the need for relationship: “*Talk about our problems. We observed that no matter how many people in the house are together, we need communication with peers. My students said they wish the school opened as soon as possible. When I asked why, they were either very bored, very overwhelmed, they would never want to see their friend's face, but they even missed him*” (female, 21-year experience).

*Academic Difficulties.* School counselors stated that among the academic difficulties experienced by their students at the onset of COVID-19 were amotivation, uncertainty regarding education, loss of learning, loss of success, effective work, academic responsibilities, adaptation to distance education, and time management. For the difficulties regarding distance education, another participant said, “*They had difficulty concentrating on the lessons during the distance education process*” (male, 20-year experience).

##### School Counseling Services at the Onset of COVID-19

When COVID-19 started, the services offered by school counselors were discussed under three main themes: (1) services for the student, (2) services for the family, and (3) services for the teacher.

*Services for Students.* Services offered by school counselors for the student at the onset of COVID-19 included individual counseling (online), individual counseling (online), form filling (online), interview (online, face-to-face, phone), group counseling (online), peer counseling, vocational guidance, preventive counseling, and social assistance. One participant expressed her opinion about online individual counseling service as follows: “*I had 10–12 students with whom I did personal counseling every week. When this process started, we tried to make an online counseling*” (female, 17-year experience).

*Services for Families.* At the onset of COVID-19, it was observed that school counselors continued the services they had previously planned for their families, in addition to family counseling, parent meetings, parent needs analysis, online parent seminar, and home visits. One participant stated that he went for a home visit to a student who lost his father as follows: “*The father of one of our students had a heart attack during this period, he passed away. I called my student…We even came together with fellow teachers and went to the house of this student who lives in a different city for a condolence visit and support*” (male, 20-year experience).

*Services for Teachers.* It was observed that school counselors' services for teachers were limited at the onset of COVID-19. These services included consultation. The statement of a participant for the consultation service is as follows: “*We held meetings every Friday. We held a meeting as a counseling service with all teachers and administrators, which lasted about one and a half hours. We made a general weekly evaluation*” (female, 21-year experience).

##### Difficulties that Students May Encounter in Transition to Face-to-Face Education

According to the opinions of the school counselors, the expressions of the students regarding the difficulties they may experience if they start face-to-face education are divided into themes in emotional, academic, and relational ways.

*Emotional Difficulties.* According to the views of the counselors, the emotions that students may experience when they start face-to-face education included fear of contagion, loss and grief, fear of losing loved ones, rule pressure, and career anxiety. One of the participants expressed her ideas about the students experiencing grief: “*In this process, there will definitely be some of our students or teachers who lose their relatives. They may have needs regarding the support and grief process*” (female, 19-year experience).

*Academic Difficulties.* According to the opinions of the school counselors, if students start face-to-face education, the academic difficulties they may experience include education under the fear of contagion, learning losses experienced in the distance education process, weakening of the school connection, loss of motivation, adaptation to school life, working habits, and time management. A participant emphasized the difficulty of education under the fear of contamination, “*We're putting 35 students into a class for 25 students…I think the physical conditions will create a much bigger problem*” (female, 21-year experience).

*Relationship Difficulties.* According to the views of the school counselors, the relational difficulties that students may experience when they start face-to-face education were keeping distance in relationships, maintaining close relationships at a distance, and family pressure. Referring to the issue of maintaining distance in their relationships, a school counselor expressed his views as follows: “*We will have students who do not want measures such as distance, masks, and who do not want to be separated from their close friends in physical distance*” (male, 20-year experience).

##### Planned Counseling Services for the Possible Needs of Students if They Can Start Face-to-Face Education

Information on the preparations planned within the school counseling services for the needs of students that may arise in case of face-to-face education during the pandemic period was determined based on the opinions of the school counselors. When the services planned by school counselors after COVID-19 were examined, it was seen that their preparations were on the following issues: orientation, psychosocial support, empowerment of families, empowerment of teachers, needs analysis, and individual and group meetings. One of the participants expressed psychosocial support studies as, “*In this period, I think we will give a lot of space and time especially to the psychosocial support program. We will try to implement the activities in that program. Especially in the psychosocial support program published this year, there were modules in areas such as migration, trauma, and suicide. Perhaps a new module on the pandemic can be prepared and implemented*” (female, 17-year experience).

### Discussion of Study 2

#### Students' Needs (at the Beginning of COVID-19)

According to the results of the study, the students expressed the difficulties they experienced at the onset of COVID-19 as educational, cognitive, emotional, physiological, relational, and technological difficulties and those related to routines. School counselors, on the other hand, described the difficulties that their students experienced at the onset of COVID-19 as family relations, personal–social, emotional, and academic ones.

It was observed that school counselors provide services for students, families, and teachers in order to meet these needs at the onset of COVID-19. The challenges high school students experienced during COVID-19 in Turkey had similarities with the results of research conducted in different countries (Gualano et al., 2020; Zhang et al., 2020). According to WHO (2020c), sudden closure of schools had negative effects on students' health, education, and development. Some studies conducted on the general population and youth during the COVID-19 period generally focus on psychological problems and psychological symptoms (Liang et al., 2020; Xiong et al., 2020). Although not being able to go to school, staying at home and curfews are deemed appropriate in terms of public health, they can have a threatening effect on the mental health of the society. There are studies showing that the stress and anxiety levels of students increase during this period when they stay at home (Huskya et al., 2020).

Rajkumar (2020) found that the COVID-19 outbreak was associated with depression, stress, sleep disorders, and other psychological symptoms. Factors that cause students' stress, anxiety, and depressive thoughts included fears and worries about themselves and their loved ones' health, focusing problems, disruption of sleep patterns, and an increase in social anxiety due to physical distance (Son et al., 2020). In the same study, academic difficulties experienced with the sudden transition to online education processes were discussed.

The challenges caused by COVID-19 and the needs of students will also change their expectations from school counseling services. The readiness of both students and school counseling offices for this change is very valuable for the protection of the mental health of the community. In the face of such a difficulty, it may not be possible for students to manage all their difficulties on their own and cope with their difficulties in the process. Although studies have given some ideas about dealing with the stress situations experienced during the COVID-19 period, it is important to produce effective and permanent services in this regard. According to a study conducted on adolescents in China, it has been observed that positively coping with the psychological symptoms associated with COVID-19 is a protective factor (Zhang et al., 2020). At the same time, seeking help from others is among the methods used along with positive coping (Son et al., 2020).

#### Students' Needs (After COVID-19)

It has been determined that students may experience difficulties in academic, social–emotional, and behavioral areas if they start face-to-face education. School counselors, on the other hand, divided the expressions about the difficulties that students may experience if they start face-to-face education into themes, emotional, academic, and relational ones. Although the difficulties of students and school counselors with respect to the onset of COVID-19 or the period when schools reopen are expressed relatively differently, the difficulty experienced or felt by each student in either period will not be as low as in the pre-COVID-19 period. It becomes important to restructure school counseling services and to shape service models and processes for each student to cope with more intense difficulties. Considering the results of this research regarding the expectations of the students from the counseling services when they return to face-to-face education and the services that the school psychological counselors plan to provide, it is remarkable that there are no new searches and many uncertainties.

As with the onset of COVID-19, it is estimated that students will seek help from the counseling service at a limited level in the future. Among the reasons for this, it is thought that it will be difficult to provide services individually, especially due to the time limitation and inadequate number of the school counselors, and that participation in group activities to be held face-to-face or online will be limited. One of the reasons for the low participation in online trainings, in particular, is the lack of trust in telephone or online services (Son et al., 2020). For this reason, school counselors may need to advertise telephone or online services and establish the trust of students, parents, and teachers in these services. It is known that there is stigma as an obstacle for students to benefit from school counseling services (Martin, 2010). Measures should be taken to prevent stigma in online services to be provided by the school counseling office.

In school counseling services, it can be especially effective to provide students with self-management and coping skills (Son et al., 2020). However, even if the school is open from now on, it may be necessary to continue all or some of the counseling services for schools remotely (online–phone, etc.) because of time efficiency and risk of contamination. For this reason, it may be necessary to increase the quality of the services to be provided online or by phone and the trust in it. In crisis situations such as COVID-19, it is necessary to consider situations that threaten mental health, to provide preventive mental health services to all students, to provide list of rehabilitation centers to students in need, as well as to identify students who have high levels of psychological difficulties and to refer the students who cannot be supported by the school counselor to appropriate mental health services.

## Implications

COVID-19 pandemic has been shown to affect psychological symptoms, such as depression, anxiety, negative self-concept, and somatization among high school students. This finding has also been emphasized in UNICEF's (2020) report. School counselors need to be prepared to deal with these situations. In this sense, school counselors can work on online intervention programs to cope with students' psychological symptoms, such as anxiety and depression. The more mindfulness self-regulation/emotion regulation techniques are included in these intervention programs, the better the results can be. At this point, dialectical behavior therapy (DDT) prepared for adolescents can be utilized (Rathus and ve Miller, 2015; Ricard et al., 2018; Karaman, 2019). DDT can be applied in four different modules (Individual therapy, Skill training, Phone calls, Consultation team meetings). The most commonly used module with adolescents is the skill training module and consists of five skills: (1) Emotion dysregulation, (2) Interpersonal dysregulation, (3) Behavioral dysregulation, (4) Cognitive dysregulation and family conflict, and (5) Self-dysregulation (Rathus and ve Miller, 2015; Karaman, 2019). School counselors should also focus on protective factors that will eliminate psychological symptoms. School counselors, who are in contact with adolescents from various demographic backgrounds, have an important role to select interventions that will empower students and initiate and maintain interventions (Sheridan and Gutkin, 2000; Moolla and Lazarus, 2014).

Adolescents develop psychologically, cognitively, and socially in addition to biological development. In other words, adolescence is a period of intense biosocial changes. From this perspective, it is difficult for adolescents to stay away from their friends in this developmental stage. However, with online social skills training by school counselors, if skills about managing and implementing their own ideas in their social networks are provided, they can overcome this situation with the least damage because adolescents have a significant capacity to influence each other (Andrews et al., 2020). School counselors and parents should be aware of this.

Undoubtedly, one of the profession groups that have difficulties in this process is school counselors. The counselors, who carried out all activities face-to-face until the pandemic, had to carry all their services online during the pandemic outbreak. However, they did not receive such an education at universities. In addition, they had to deal with such a large student crowd alone with increased psychological symptoms. In this process, either the number of school counselors should be increased or other school staff should be included in providing consultancy services during the outbreak. For example, school counselors can focus on psychological symptoms by assigning other teachers to monitor students who have difficulties in academic development. Otherwise, school counselors will come to the point of exhaustion (Luthar and Mendes, 2020).

In summary, considering the difficulties experienced by students and teachers in this process, all plans should be adjusted according to online consultancy in situations that may occur in the future such as pandemics or require online consultation. It is important for universities to plan accordingly and for public institutions to support this structuring.

## Limitations and Suggestions for Future Studies

The study was conducted on an exceptional time. When we collected data, the quarantine restrictions were strictly enforced. There was uncertainty about where the pandemic was heading, and it was not clear how long it would last. Vaccine studies had progressed significantly but did not come close to the end. Therefore, the results should be evaluated taking these into account. In other words, if the study is repeated with a similar group now, different or reverse results may be obtained because the conditions are not the same. In addition, the cross-sectional nature of the Study 1 prevents concerns about causality. Last, although the current study has statistically significant and meaningful results, it should be evaluated taking into account the low response rate, the cross-sectional characteristics of the study, the data collection period, and the group of participants. Hence, Study 1 should be taken as a preliminary research.

One limitation that may be related to Study 2 is that the interviews were made remotely and by phone and not face to face. Therefore, non-verbal bodily reactions of the interviewees were ignored in our study. Last, the current study is a preliminary study, and due to the nature of qualitative studies, we cannot generalize our findings to the general population. The study should be repeated in order to reach more information about this issue or to reach general judgments.

Counselors working with adolescents should be investigated for their capacity to help students after this trauma and what kind of psychoeducation/training programs they need in this regard. In addition, research is needed to increase the level of well-being of students and teachers. In addition to this research, which examines the individual psychological effects of the pandemic period and its aftermath on students, the research can be expanded by adding all environmental factors (microsystem, mesosystem, exosystem, and macrosystem), or this study can be repeated over different age groups (university students, counseling centers).

## Conclusion

Contemporary and developed societies aim for students to lead a better quality of life by providing their cognitive, social, emotional, moral, and behavioral development with the contents in their education systems. From this point of view, it has been obvious that schools have a determining effect on the quality of life of students. It is inevitable for schools, which have such a determining effect on the development of individuals, to undertake a function that facilitates coping with the difficulties that students experience and may experience in the process and the problems that may arise due to these difficulties. Student personality services to be prepared and implemented by school counseling services are vital for schools to fulfill this function. It has become inevitable for schools to make plans regarding situations such as (a) the possibility that COVID-19 pandemic may continue for a long time and (b) COVID-19 pandemic can be brought under control in a short time and education can be started again in its old normal order. In order for school counseling services to provide the psychological support and other assistance services needed in both cases, sufficient numbers of well-equipped and competent school counselors are needed.

## Data Availability Statement

The raw data supporting the conclusions of this article will be made available by the authors, without undue reservation.

## Ethics Statement

The studies involving human participants were reviewed and approved by Kilis 7 Aralik University. Written informed consent to participate in this study was provided by the participants' legal guardian/next of kin.

## Author Contributions

MAK conceived the study and ran the analyses for Study 1 and wrote methods, results, and discussion for Study 1. HE ran the analyses, and wrote methods, results, and discussion for Study 2. İHT wrote the implications, limitations, and conclusion. RA wrote the introduction. All authors contributed to the article and approved the submitted version.

## Conflict of Interest

The authors declare that the research was conducted in the absence of any commercial or financial relationships that could be construed as a potential conflict of interest.
